# Editorial: Advancements in dietary supplements: enhancing sport performance and recovery

**DOI:** 10.3389/fspor.2025.1703153

**Published:** 2025-10-10

**Authors:** Mark E. T. Willems, Laurel M. Wentz, David C. Nieman

**Affiliations:** ^1^ University of Chichester, Chichester, United Kingdom; ^2^ Appalachian State University, Boone, NC, United States

**Keywords:** dietary supplements, sports nutrition, athletes, sports performance, exercise recovery

**Editorial on the Research Topic**
Advancements in dietary supplements: enhancing sport performance and recovery

Dietary supplement consumption is common among athletes and recreationally active individuals. The number of studies listed in PubMed, the reputable database from the National Library of Medicine (United States) that include “dietary supplements” [Title/Abstract] OR “dietary supplement” [Title/Abstract] will soon exceed 20,000 records. However, very few dietary supplements have been scientifically linked to enhanced athletic performance or recovery. Those with strong scientific evidence include creatine, caffeine, β-alanine, protein, and nitrate (1). The majority of other supplements have mixed scientific support or remain unproven and may even be risky. A food-first approach based on a well-balanced, nutrient-rich diet is recommended for the majority of athletes and exercisers (2). A sports nutrition professional should be contacted for guidance before taking any supplement.

The Research Topic “*Advancements in dietary supplements: enhancing sport performance and recovery*” features one review and four experimental studies that contribute to our understanding of the ergogenic potential and application of dietary supplements in diverse cohorts. In the article “*Emerging evidence of Urolithin A in sports nutrition: bridging preclinical findings to athletic applications*”, Wang and Yu provided information warranting future research on Urolithin A. Urolithin A is a gut-derived metabolite from ellagatins and ellagic acid. These polyphenols are present in foods such as walnuts and pomegranates. The inter-individual variability in urolithin availability from natural sources is age-dependent, with 10% of individuals being non-producers independent of age (3). Nevertheless, Urolithin A may be applied as a dietary and sports nutrition supplement due to its potential antioxidant effects, which may reduce muscle fatigue and support cardiovascular function. Based on the studies covered in the review by Wang and Yu, recommendations were provided for a dosing strategy for Urolithin A to be used in future exercise studies.

The remaining four studies in the Research Topic are experimental. The study by Zhu et al. “*Effects of progressive versus consistent dose of caffeine ingestion on volleyball players’ exercise performance adaptations following plyometric jump training*” examined different caffeine dosing strategies. The majority of dietary supplement studies use a constant dose to examine ergogenic potential. Lara et al. (4) suggested that increased caffeine tolerance occurs with a constant dose (3 mg/kg body weight) over 20 days. Zhu et al. observed that a progressive dose of caffeine (3 mg/kg body weight to 6 mg/kg body weight) over 4 weeks combined with plyometric jump training in male Chinese volleyball players was as effective on high-intensity exercise and agility observations as a constant high dose of 6 mg/kg body weight. The combined use of dietary supplements is likely common although the majority of the studies on the prevalence of dietary supplements do not report on combined use (5). Abdioglu et al. examined the effects of the combined use of carbohydrate gels and caffeinated chewing gum on male junior Turkish tennis players. The effects of the combined use of ground strokes were better than those of the control and placebo chewing gums, with lower RPE values during the game-simulated tennis tasks. While no difference in performance outcomes was identified in this study, the combined supplementation of carbohydrate gels and caffeinated gum may reduce fatigue with potential effects on performance throughout the tennis season. Understanding the effectiveness of dietary supplements requires recognizing inter- and intra-individual responses (6). Niknam et al. examined individual responses to the intake of purple grape juice (10 ml/kg of body mass) in a cohort of 22 elite male Iranian soccer players under 20 years of age. Their findings revealed that purple grape juice had more participants responding in a meaningful way during the exercise required during the 30–15 Intermittent Fitness Test (∼74%) than during the recovery measurement of a standing long jump after exercise (54%). Studies addressing inter-individual responses, including the one by Niknam et al., contribute to the personalized use of dietary supplements. Finally, the article by Wang et al. examined the effect of dietary nitrate (beetroot) in male Chinese college bodybuilders. Beetroot is a popular dietary supplement (7) and is known to be ergogenic in endurance activities. Wang et al. observed enhanced endurance in the third round of repeated isometric circuit endurance tests (four rounds) with different muscle groups. Endurance improved by 14.9%, 25.4%, and 25.2% for the elbow flexors, forearm muscles, and knee extensor muscles, respectively. These findings suggest that a level of fatigue is required for beetroot to be effective, which could affect the recovery process between isometric tasks in bodybuilding.

Numerous factors influence the effectiveness of dietary supplements on exercise performance and recovery, including age, training status, skill level, sport, sex, ethnicity, habitual diet, and gut microbiome, among others. Not to mention, new compounds will make advancing the field through experimental studies a challenging undertaking. The articles in this Special Issue “*Advancements in Dietary Supplements: Enhancing Sport Performance and Recovery*” are modest considering the vastness of unexplored issues.
